# Identification of differentially expressed genes at the single-cell level and prognosis prediction through bulk RNA sequencing data in breast cancer

**DOI:** 10.3389/fgene.2022.979829

**Published:** 2022-09-16

**Authors:** Hanghang Chen, Tian Tian, Haihua Luo, Yong Jiang

**Affiliations:** Guangdong Provincial Key Laboratory of Proteomics, State Key Laboratory of Organ Failure Research, Department of Pathophysiology, School of Basic Medical Sciences, Southern Medical University, Guangzhou, China

**Keywords:** breast cancer, single-cell, prognosis, tumor mutational burden, immune infiltration, bulk RNA-seq, transcriptomics

## Abstract

**Background:** The invention and development of single-cell technologies have contributed a lot to the understanding of tumor heterogeneity. The objective of this research was to investigate the differentially expressed genes (DEGs) between normal and tumor cells at the single-cell level and explore the clinical application of these genes with bulk RNA-sequencing data in breast cancer.

**Methods:** We collected single-cell, bulk RNA sequencing (RNA-seq) and microarray data from two public databases. Through single-cell analysis of 23,909 mammary gland cells from seven healthy donors and 33,138 tumor cells from seven breast cancer patients, cell type-specific DEGs between normal and tumor cells were identified. With these genes and the bulk RNA-seq data, we developed a prognostic signature and validated the efficacy in two independent cohorts. We also explored the differences of immune infiltration and tumor mutational burden (TMB) between the different risk groups.

**Results:** A total of 6,175 cell-type-specific DEGs were obtained through the single-cell analysis between normal and tumor cells in breast cancer, of which 1,768 genes intersected with the bulk RNA-seq data. An 18-gene signature was constructed to assess the outcomes in breast cancer patients. The efficacy of the signature was notably prominent in two independent cohorts. The low-risk group showed higher immune infiltration and lower TMB. Among the 18 genes in the signature, 16 were also differentially expressed in the bulk RNA-seq dataset.

**Conclusion:** Cell-type-specific DEGs between normal and tumor cells were identified through single-cell transcriptome data. The signature constructed with these DEGs could stratify patients efficiently. The signature was also closely correlated with immune infiltration and TMB. Nearly all the genes in the signature were also differentially expressed at the bulk RNA-seq level.

## Introduction

Breast cancer is the most prevalent malignancy in females worldwide and accounted for about 30 % of new cancer cases in 2020 (Siegel et al., 2020). Currently, it is not precise enough to predict the prognosis of patients by clinicians with just clinical stage information. Traditionally, molecular subtypes based on estrogen receptor (ER) expression, progesterone receptor (PR) expression, and human epidermal growth factor receptor-2 (HER2) amplification could provide some references for treatment decision-making, but they do not capture the essential characteristics of tumors (Pusztai et al., 2006). In this regard, novel methods and deep understanding are warranted to facilitate personalized medicine.

Malignancy is a genetic disease characterized by an aberrant expression of multiple genes and accumulating gene mutations (Lengauer et al., 1998). The key oncogenic transformation which breaks the balance between tumorigenesis and immune clearance has not been elucidated. Moreover, the heterogeneity of malignancies is also attributed to various degrees of differentiation, proliferation, and invasiveness in different patients (Feng et al., 2018). Therefore, it is crucial to identify the differentially expressed genes (DEGs) between normal and tumor tissues which might be the cardinal genes in the multistep progression of tumors. The microarray (Abd-Elnaby et al., 2021) and next-generation sequencing (NGS) (Hong et al., 2020) technologies provide convenient approaches for gene expression profiling. These technologies have proven beneficial for risk prediction, patient stratification, and mechanism investigation (Chen et al., 2022).

A tumor microenvironment contains multiple components or various cells, such as cancer cells and immune cells (Li et al., 2007; Shi et al., 2020). Numerous bulk RNA-seq analyses provided hints about the differences between a normal and tumor microenvironment (Wang et al., 2020; Lu et al., 2021; Chen et al., 2022). However, bulk RNA-seq only provides average expression profiles which may lead to misunderstanding of the cardinal features. The invention and development of single-cell RNA-seq (scRNA-seq) offer cell-type-specific insights (Chen et al., 2019a; Chen et al., 2019b). The genes which drive the tumor progression forward or inhibit the initiation may be investigated at a single-cell resolution.

Given that bulk RNA-seq analyses could provide correlations between transcriptome profiles and clinical information and cell type-specific insights from scRNA-seq, it is of critical importance to bridge the analyses of single-cell and bulk RNA-seq data. There are such increasing efforts from different research communities who generated single-cell and bulk data using different methods (Bao et al., 2021; Chen et al., 2021; Li et al., 2021). Nonetheless, insights about cell-type-specific DEGs are still deficient. Hereby, in this study, we attempted to afford a means to integrate data from these two dimensions. Through the integrative analyses, we also provided a new insight into the study of neoplasia and developed a gene signature to facilitate the prognosis prediction for breast cancer. Moreover, all of the DEGs, except for two, in the signature expressed differentially between tumor and normal tissues in the bulk RNA-seq data, which could support the importance of the genes.

## Materials and methods

### Data collection

The scRNA-seq data on 23,909 mammary gland cells from seven healthy donors and 33,138 tumor cells from two ER+, two HER2 amplifications, one PR+, and two triple-negative (TN) breast cancer (BC) patients were downloaded from the Gene Expression Omnibus (GEO) database (https://www.ncbi.nlm.nih.gov/geo/, GSE161529) (Pal et al., 2021).

The bulk RNA-seq data on female breast cancer patients were collected from The Cancer Genome Atlas (TCGA) database (Tomczak et al., 2015). The microarray data on 532 breast cancer patients were collected from the GEO database (GSE20685, GSE20711, and GSE88770) (Dedeurwaerder et al., 2011; Kao et al., 2011; Metzger-Filho et al., 2013). They were removed of the batch effects using the “sva” R package (Powell et al., 2018) and merged as the GEO cohort.

We downloaded the reference file for the gene set variation analysis (GSVA) from a website (http://www.gsea-msigdb.org/).

### Single-cell RNA-seq data analyses

We conducted single-cell RNA-seq data analyses according to the pipeline of the “Seurat” R package (Butler et al., 2018). Unqualified cells (the gene counts per cell≤300 and percentage of mitochondrial genes per cell≥5) were removed. We normalized the expression matrix of all cells with the “LogNormalize” method and then identified 2,000 highly variable genes among the cells *via* the “vst” method.

We conducted principal component analysis (PCA) to reduce the dimensions of the data. With the help of an elbow plot, we selected the top 19 principal components to cluster cells with the marker genes. We utilized the “harmony” algorithm to integrate the data from different samples (Korsunsky et al., 2019). We used the “SingleR” R package to annotate the cell types (Aran et al., 2019) and validated the annotation *via* cell markers from a previous study (Aran et al., 2017). We displayed a landscape of all the cells using the uniform manifold approximation and projection (UMAP). Finally, we identified the seven clusters of DEGs between normal and tumor cells in the seven cell types, respectively (|log fold change (logFC)| > 0.5 and *p*-value < 0.05).

To investigate the differences of the biological pathways in each individual cell type, we also conducted GSVA scores of the 50 hallmark pathways in normal and cancer cells (Hanzelmann et al., 2013).

### Construction and validation of a prognostic signature

In the two bulk datasets, we normalized the RNA expression levels with the “limma” and “sva” R packages (Powell et al., 2018). We randomly assigned 70 percent of patients in TCGA dataset to the training cohort, and the rest of the patients were assigned to the test cohort. With the “survival” R package, we conducted univariate Cox regression to obtain prognosis-related DEGs in the training cohort. With these genes, we developed a prognostic signature including several genes with the least absolute shrinkage and selection operator (LASSO) regression method in the “glmnet” R package. The risk score of each patient was calculated with ∑ (expression of gene*i* ∗ β*i*), where β was the coefficient of each gene.

The median risk score of the training cohort assisted the stratification of patients in the training, test, and GEO cohorts separately into low-risk and high-risk groups. We compared the overall survival (OS) differences between different risk groups by the Kaplan–Meier (KM) method in the “survival” R package.

Patients in TCGA cohort were divided into stage I–II and III–IV subgroups. We also validated the efficacy of the signature in the two subgroups, respectively, using the KM method.

We further validated the efficacy of the signature by conducting 3-, 5-, and 8-year ROC (receiver operating characteristic curve) analyses using the “time-ROC” R package in the training, test, and GEO cohorts separately.

### Nomogram model construction and validation

To improve the efficacy of the prediction, we incorporated clinical factors (age and stage) and constructed a nomogram model *via* the “rms” R package in TCGA cohort.

To validate the efficacy of the nomogram, we plotted 3-, 5-, and 8-year ROC curves to assess the efficacy of the nomogram. We also demonstrated the efficacy of the nomogram through a calibration plot.

### Gene set enrichment analysis and immune infiltration differences

To figure out the implications of different risk scores, we performed GSEA with the clusterProfiler R package (Wu et al., 2021) to explore the biological pathways differentially enriched in the high- and low-risk groups separately.

Considering the differences of immune-related pathways through GSEA, we calculated the immune infiltration score of every sample of TCGA dataset *via* single-sample gene set enrichment analysis (ssGSEA) (Hanzelmann et al., 2013). Subsequently, we compared the differences of the immune cells and pathways between different risk groups. We also utilized the “estimate” R package to evaluate the immune infiltration (ImmuneScore, StromalScore, and ESTIMATEScore) (Galon et al., 2012) and compared the differences in immune infiltration between different risk groups.

### Tumor mutational burden and tumor-immune dysfunction and exclusion differences

Considering the DEGs between normal and tumor cells might indicate different accumulations of mutations. Tumor mutational burden (TMB) could be a common indicator of malignancy for tumors. We calculated the TMB of each sample in TCGA dataset by Varscan software (Koboldt et al., 2012) and compared the difference between the high- and low-risk groups.

Tumor immune dysfunction and exclusion (TIDE) could predict patient responses to anti-PD1 and anti-CTLA4 therapies. We calculated the TIDE scores from the website (http://tide.dfci.harvard.edu/) (Fu et al., 2020) and compared the differences between different risk groups.

Investigation of the expression of the genes in single-cell and bulk RNA-seq data

To validate the signature genes in bulk RNA-seq data, we compared the expression levels of the 18 genes in the signature between normal and tumor samples in TCGA dataset. We also compared the expression levels of the 18 signature genes between normal and tumor cells in the seven cell types separately.

To understand the importance of these genes and investigate the differential expression in the different developmental states of cells, we performed a pseudotime analysis with the “Monocle” R package (version 2.22) (Qiu et al., 2017).

### Statistical analysis

The Wilcoxon test was utilized to compare the differences between the two groups. Correlations between variables were calculated by the Spearman test. All statistical analyses were conducted with R software (v4.1.3), and *p* < 0.05 was considered statistically significant. We utilized “set.seed” function in our codes to guarantee that the analysis was reproducible.

## Results

### DEGs between normal and tumor cells through analyses of scRNA-seq data

**FIGURE 1:**
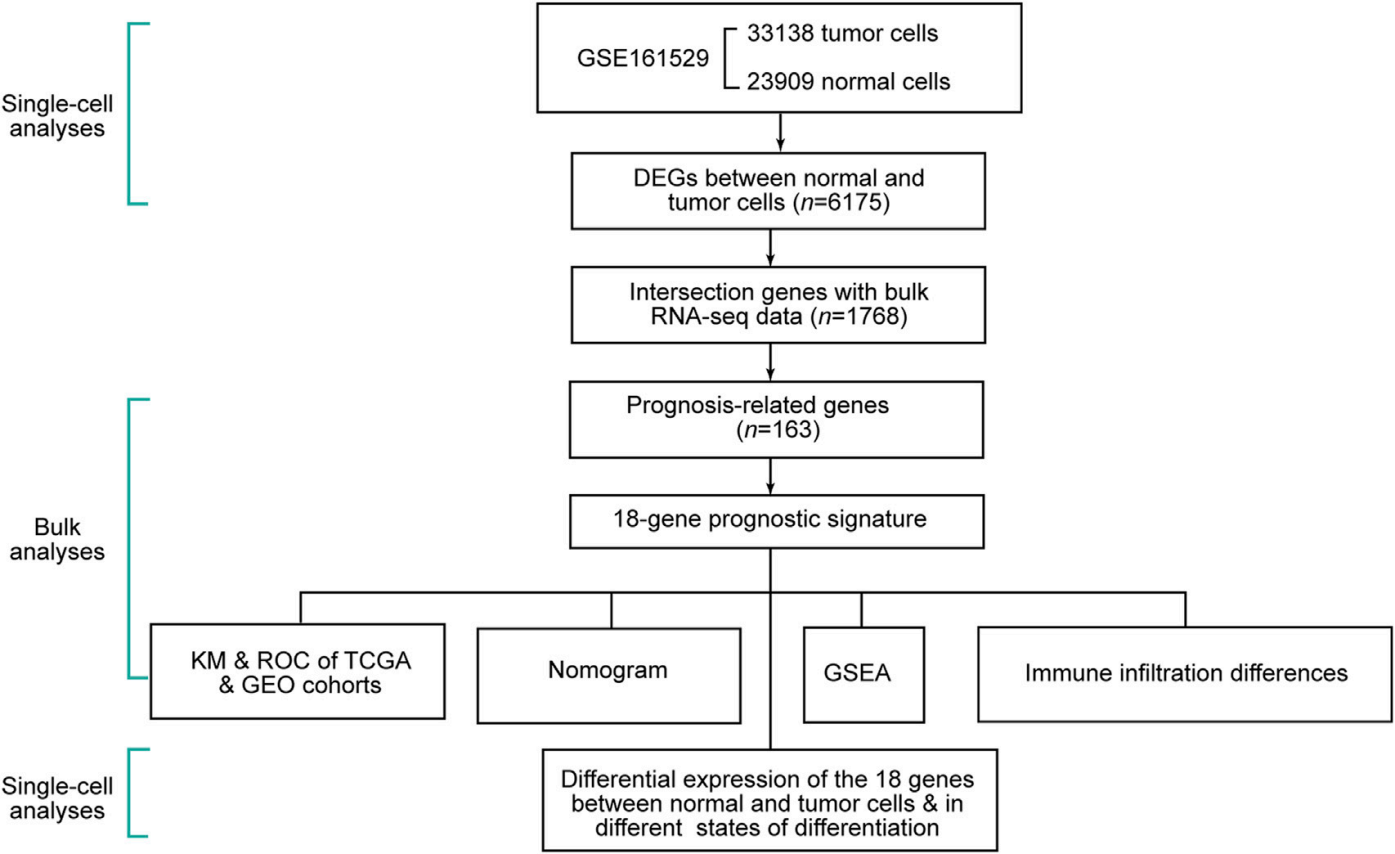
Schematic diagram of this study.

The schematic diagram of this study is shown in Figure 1. We removed cells with less than 300 gene counts and more than 5 % mitochondrial gene counts (Supplementary Figure S1A). We defined the top 2,000 DEGs among the cells as variable genes, and the top 10 were labeled (Supplementary Figure S1B). With the expression of the 2,000 variable genes, we conducted PCA to reduce the dimensions and displayed all cells in the top two dimensions (Supplementary Figure S1C). The elbow plot helped us select 1–19 PCs in the downstream analyses (Supplementary Figure S1D).

**FIGURE 2:**
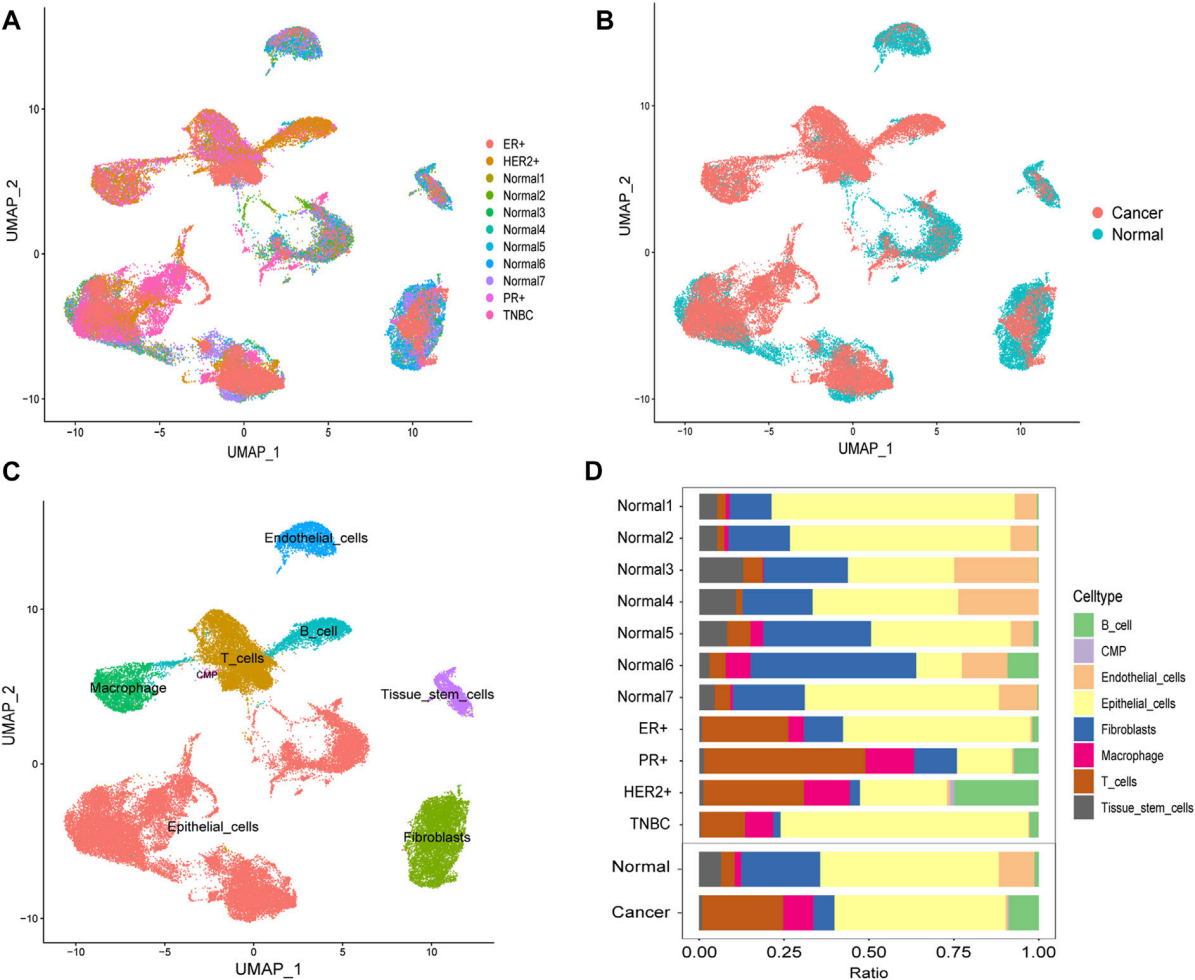
Cell-type annotation and cell ratios. **(A)** Uniform manifold approximation and projection (UMAP) displayed the cell distribution of normal and different subtypes of breast cancer. **(B)** Cell distribution of normal and cancer cells. **(C)** Eight cell types were annotated and labeled. **(D)** Differences of the cell ratios between normal and tumor samples.

Through the DEGs between cells, we classified all the cells into 22 clusters. We displayed the cell distribution of normal and different subtypes of breast cancer (Figure 2A). The distribution of normal and cancer cells (Figure 2B) could demonstrate that the integration of different samples was efficient. We annotated eight cell types with the “SingleR” R package and labeled them in Figure 2C. To validate the accuracy of cell annotation, we summarized some marker genes and displayed their expressions (Supplementary Figure S2).

**FIGURE 3:**
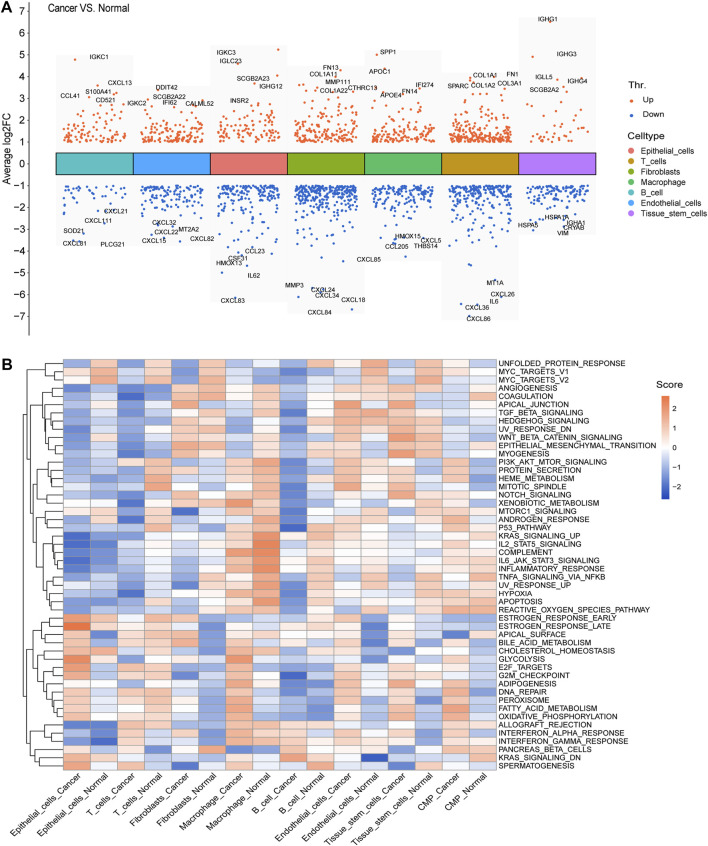
DEGs and GSVA. **(A)** Seven clusters of significant DEGs between normal and tumor cells in the seven cell types were separately identified, and the top five upregulated and downregulated DEGs of each cluster were labeled. **(B)** Average gene set enrichment analysis (GSVA) scores of each cell type in normal and cancer cells, respectively, displayed the scores of the 50 hallmark pathways.

We calculated and displayed the cell ratios of normal and tumor samples, respectively, in Figure 2D. There were only three common myeloid progenitor (CMP) cells in cancer samples, and thus, this cell type was neglected when we calculated the DEGs. Therefore, seven clusters of significant cell-type-specific DEGs between normal and tumor cells in seven cell types are separately displayed in Figure 3A and listed in Supplementary Table S1.

To facilitate the understanding of the functional differences between normal and cancer cells, we calculated and displayed the average GSVA scores of 50 hallmark pathways for each cell type in normal and cancer cells, respectively (Figure 3B).

### Identification of an 18-gene signature and validation

**FIGURE 4:**
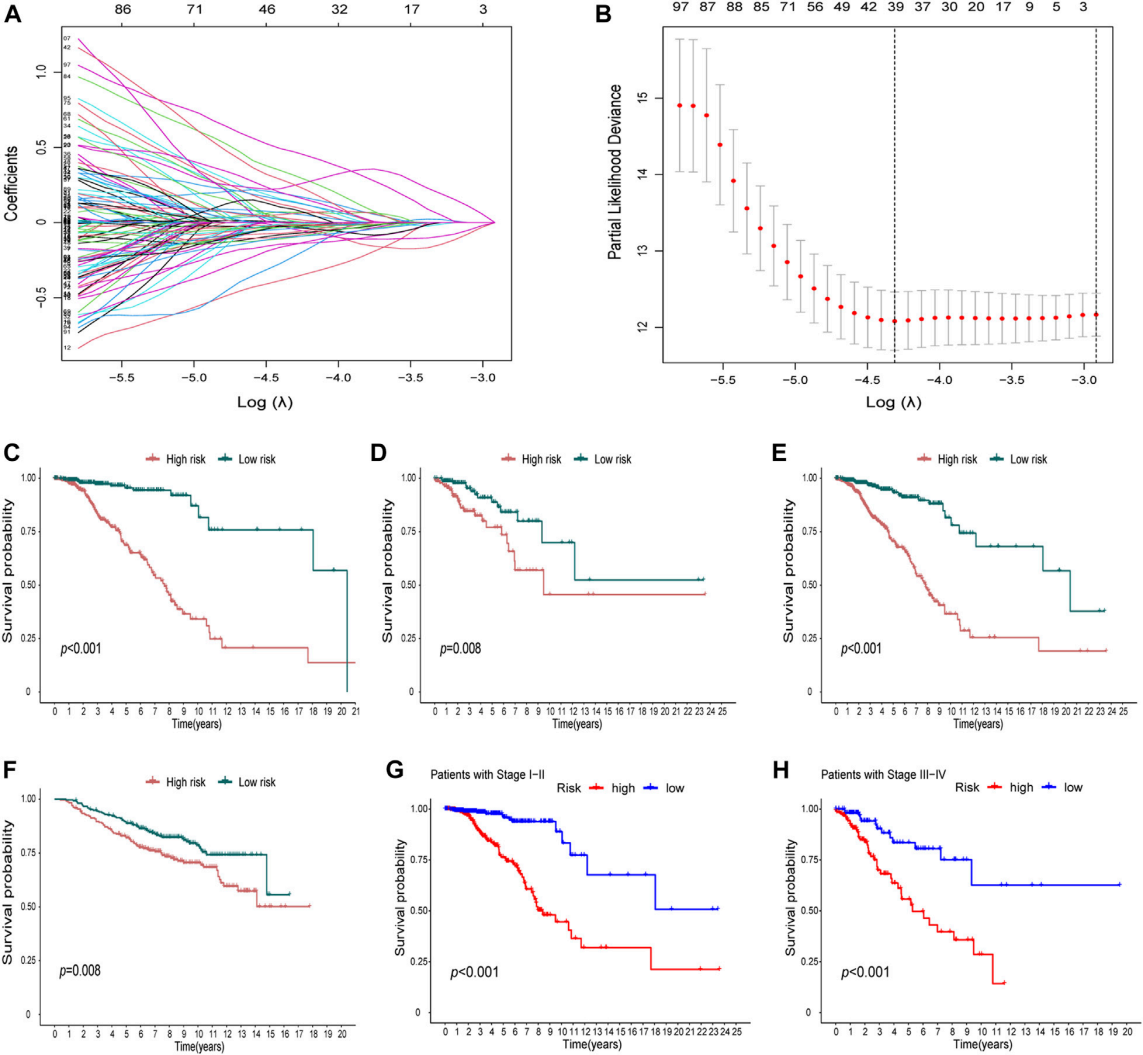
Identification of a prognostic signature in the training cohort and validation cohort. **(A-B)** At the bulk level, a signature including 18 genes was developed based on the prognostic DEGs and the optimal λ through the least absolute shrinkage and selection operator (LASSO) regression method. **(C-F)** KM curves displayed a significantly better overall survival (OS) of the low-risk group in the training cohort **(C)**, the test cohort **(D)**, the whole TCGA cohort, **(E)** and the GEO cohort **(F)**. **(G**–**H)** In the stage I–II **(G)** and stage III–IV **(H)** subgroups of TCGA cohort, OS of the low-risk group is also better than that of the high-risk group.

In the DEGs, 163 genes were prognostic in the training cohort through univariate Cox regression and are listed Supplementary Table S2. Through the LASSO regression, a signature comprising 18 genes was developed based on the optimal λ (Figures 4A, B). Table 1 lists the 18 genes and their coefficients which could be used to calculate the risk score of every sample.

**TABLE 1:** Genes in the signature and their coefficients calculated by using the LASSO algorithm.

Gene	Coefficient
*AIMP2*	0.950789
*SNX3*	0.790702
*ARID1B*	0.736265
*ZMAT3*	0.664705
*OR51E1*	0.450027
*C8orf33*	0.411607
*TCEAL8*	0.393024
*ACSL1*	0.331655
*IGFBP5*	0.135417
*KRT15*	−0.07247
*RBP1*	−0.17784
*PIGR*	−0.22442
*PLGRKT*	−0.26485
*LEF1*	−0.27798
*FAM118A*	−0.34295
*BCL2A1*	−0.39692
*ANXA5*	−0.62069
*NGDN*	−0.76176

The KM curves displayed a significantly better OS of the low-risk group in the training cohort (Figure 4C). We also showed that in the test cohort (Figure 4D), the whole TCGA cohort (Figure 4E), and the GEO cohort (Figure 4F), the signature could stratify patients into different risks well. In the stage I–II and stage III–IV subgroups of TCGA cohort, the OS of the low-risk group was also better than that of the high-risk group (Figures 4G,H).

**FIGURE 5:**
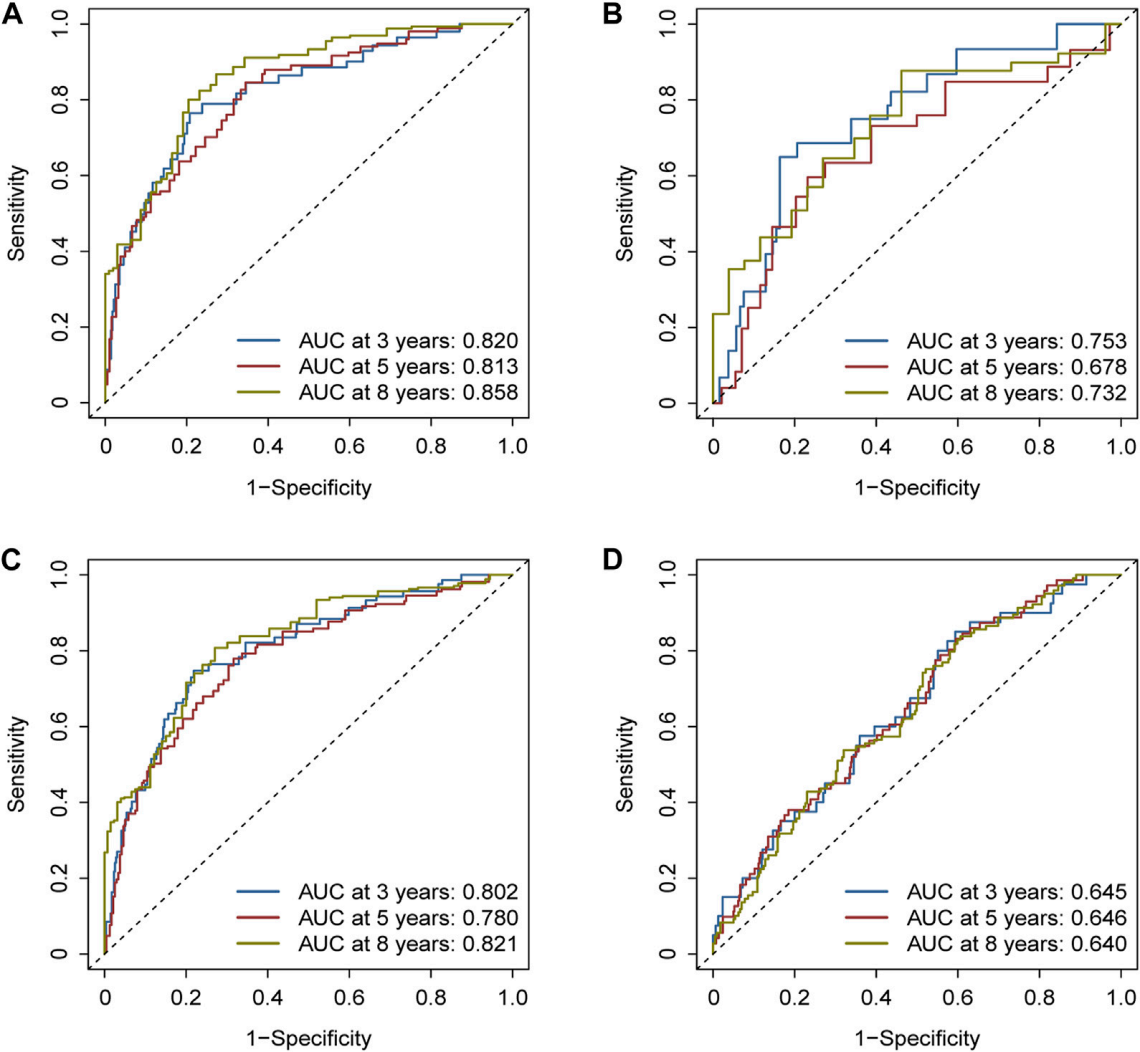
Assessment of the efficacy of the risk signature. **(A)** Areas under the ROC curves (AUCs) were 0.820, 0.813, and 0.858 for 3-, 5-, and 8-year survival, respectively, in the training cohort. **(B)** Likewise, AUCs were 0.753, 0.678, and 0.732 in the test cohort. **(C)** Likewise, AUCs were 0.802, 0.780, and 0.821 in the whole TCGA cohort. **(D)** Likewise, AUCs were 0.645, 0.646, and 0.640 in the GEO cohort.

The areas under the ROC curves (AUCs) were 0.820, 0.813, and 0.858 for the 3-, 5-, and 8-year survivals, respectively, in the training cohort (Figure 5A). Separately, AUCs were 0.753, 0.678, and 0.732 for the 3-, 5-, and 8-year survival for the test cohort (Figure 5B) and 0.802, 0.780, and 0.821 for the whole TCGA cohort (Figure 5C). AUCs were 0.645, 0.646, and 0.640 for the GEO cohort (Figure 5D). All the results aforementioned demonstrated that the prognostic signature was significant for breast cancer.

### Nomogram construction and validation

**FIGURE 6:**
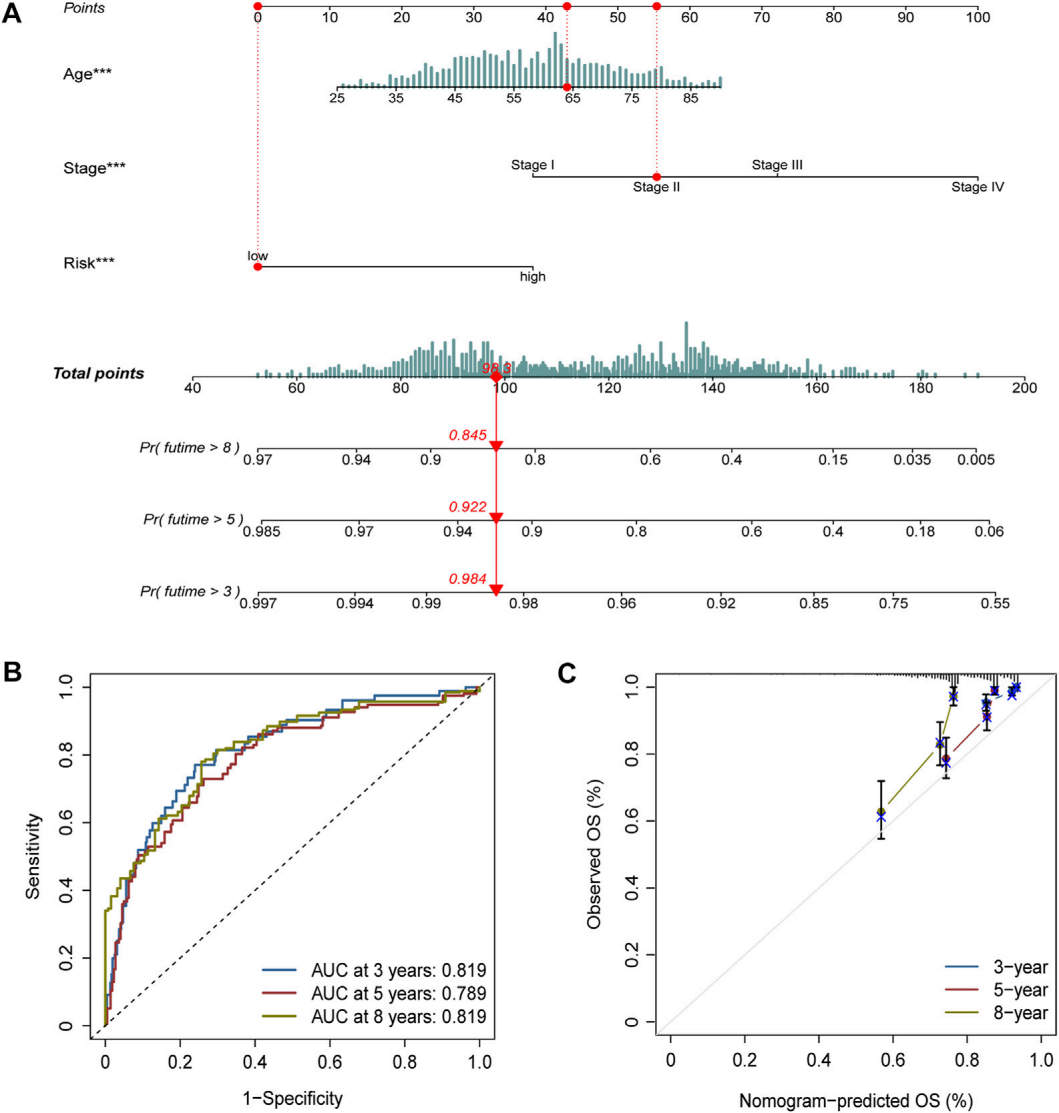
Nomogram construction and validation. **(A)** Age, stage, and risk score were included to construct a nomogram. The point of age, stage, and risk score was calculated with reference to the nomogram, and the total points could help determine the outcomes. **(B)** Efficacy of the nomogram was also assessed with ROC curves, and the AUC was separately 0.819, 0.789, and 0.819 for 3-, 5-, and 8-year survival. **(C)** Calibration plot demonstrated the consistency of the nomogram with true clinical outcomes.

Age, stage, and risk score were incorporated into a nomogram to improve the efficacy and facilitate the clinical application of the signature (Figure 6A). The points of age, stage, and risk score were calculated with reference to the nomogram, and the total points could help determine the outcomes.

The efficacy of the nomogram was also assessed with ROC curves, and the AUC values were 0.819, 0.789, and 0.819 for 3-, 5-, and 8-year survival (Figure 6B). The calibration plot demonstrated the consistency of the nomogram with true clinical outcomes (Figure 6C).

### Gene set enrichment analysis differences between different groups

**FIGURE 7:**
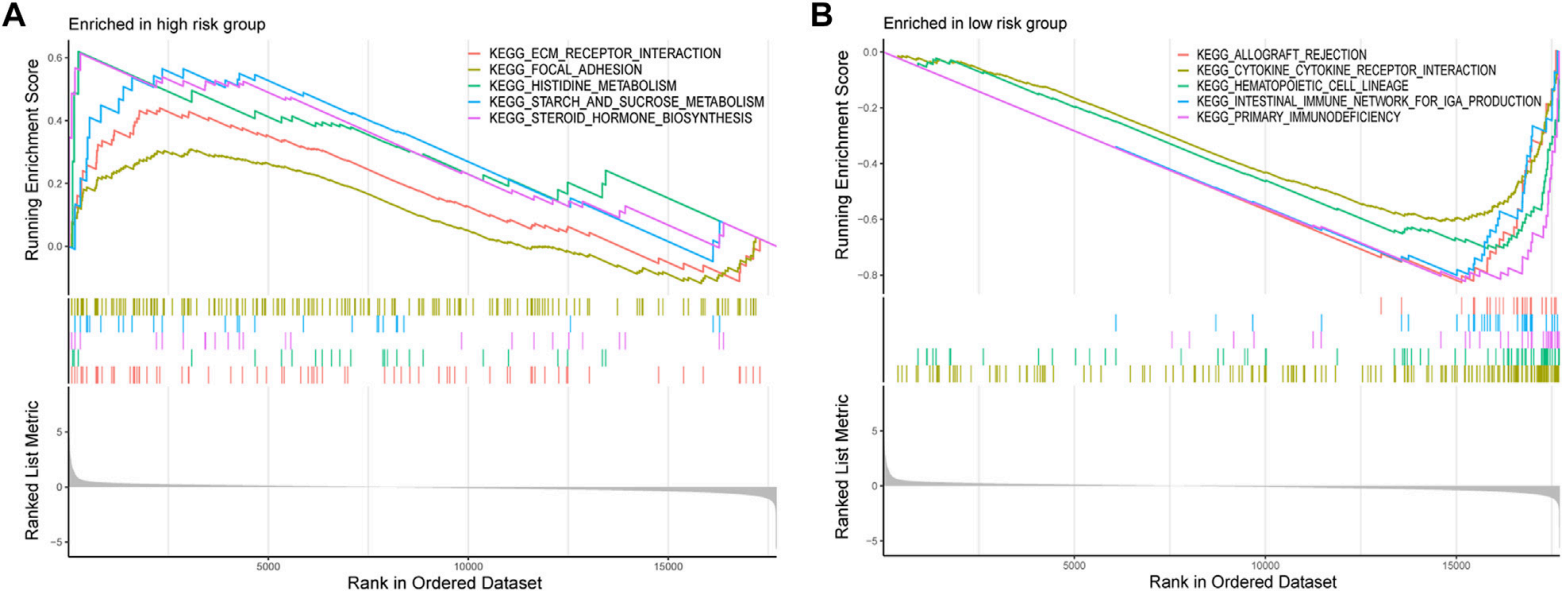
GSEA was conducted to investigate the differentially enriched pathways between high-risk and low-risk groups. **(A)** High-risk group harbored higher metabolism-related pathways. **(B)** Immune-related pathways enriched in the low-risk group.

GSEA was conducted to investigate the differentially enriched pathways between different risk groups. The high-risk group harbored higher metabolism-related pathways such as histidine metabolism and starch and sucrose metabolisms which indicated the exuberant metabolism in the high-risk samples (Figure 7A). Immune-related pathways were enriched in the low-risk group such as allograft rejection, intestinal immune network for IgA production, and primary immunodeficiency, which might provide hints that immune infiltration occurred more in low-risk samples (Figure 7B).

### Immune infiltration differences between different groups

**FIGURE 8:**
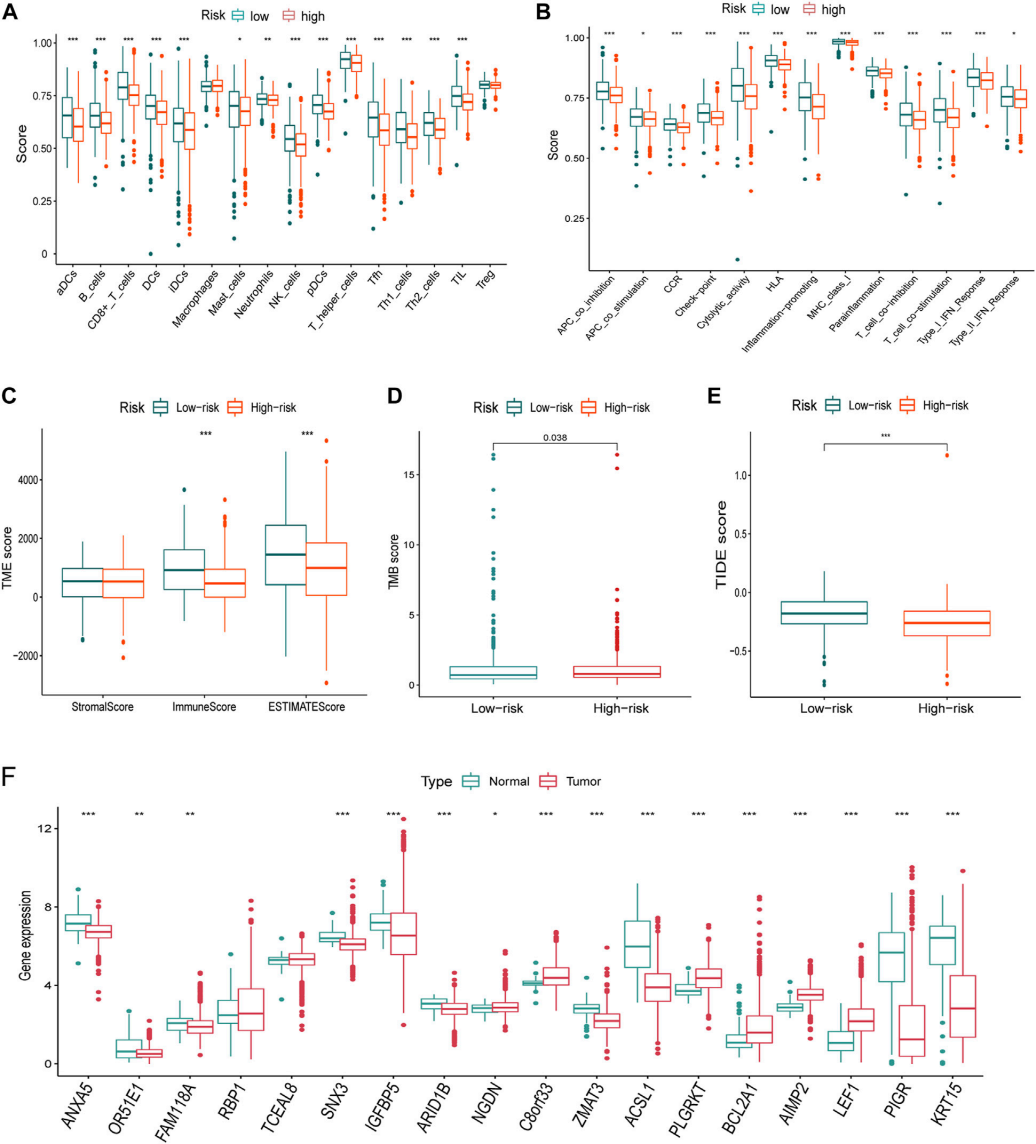
Immune infiltration, TMB, TIDE differences, and the expression of the signature genes in TCGA dataset. **(A-B)** Nearly all immune cells **(A)** and activities **(B)** were higher in the low-risk group. **(C)** ImmuneScore using the “ESTIMATE” algorithm also supported the immune infiltration advantage in the low-risk group. **(D)** TMB was higher in the high-risk group than that of the low-risk group. **(E)** Higher TIDE score indicated poorer responses to the immune checkpoint blockades, and patients in the high-risk group were more sensitive. **(F)** Nearly all of the signature genes were also differentially expressed between normal and tumor tissues in TCGA dataset (*<0.05, **: *p* < 0.01, and ***: *p* < 0.001).

Considering the differences of the immune-related pathways through GSEA, we also investigated the immune infiltration differences between different risk groups. Immune infiltration scores of every sample in the TCGA dataset are listed in Supplementary Table S3. Virtually all immune cells (Figure 8A) and activities (Figure 8B) were higher in the low-risk group. Furthermore, the “ESTIMATE” algorithm also supported the immune infiltration advantage in the low-risk group (Figure 8C).

### Tumor mutational burden differences between different groups

The TMB score of every breast cancer patient studied from TCGA database is listed in Table S4. TMB is higher in the high-risk group than that of the low-risk group (Figure 8E). A higher TIDE score indicated poorer responses to immune checkpoint blockades, and patients in the high-risk group were more sensitive.

### Differences of gene expression and developmental states

**FIGURE 9:**
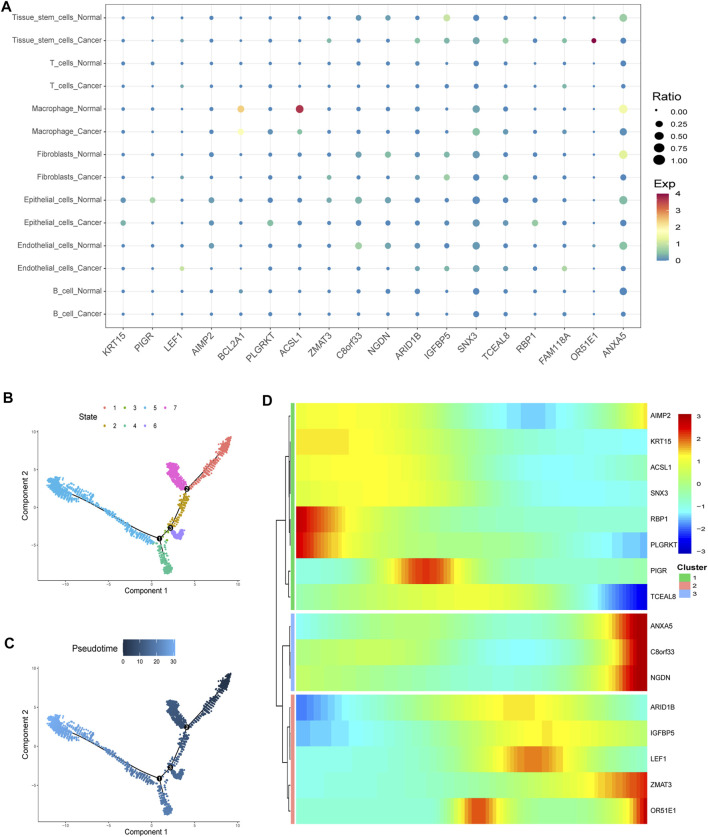
Gene expression in particular cell types and differences. **(A)** Expression levels and ratios in the seven corresponding cell type of the signature genes were displayed in normal and cancer cells, respectively (EXP: expression level). **(B**–**C)** Cells were ordered along different trajectories which meant different developmental states **(B)** or pseudotimes **(C)**. **(D)** Genes in the signature were differentially expressed in different developmental states, and they were classified into three clusters based on their expression profiles.

Virtually, all signature genes were also differentially expressed between the normal and tumor tissues in TCGA dataset (Figure 8F). The expression levels and ratios in the corresponding cell types of signature genes were shown for normal and cancer cells, respectively (Figure 9A). Cells were ordered along different trajectories, which meant different developmental states (Figure 9B) or pseudotimes (Figure 9C). Genes in the signature were differentially expressed in different developmental states (Figure 9D), and they were classified into three clusters based on their expression profiles.

## Discussion

Tumor cells are endowed with multiple abilities through the differential expression of many genes (Hanahan and Weinberg, 2011). Hence, it would make sense to identify tumor-promoting genes that induce and help sustain tumorigenesis and can thereby serve as potential targets for vaccine designs. Furthermore, tumor-antagonizing genes could advance our understanding of tumor escape from immune recognition and elimination (Bates et al., 2018). Increasing evidence from studies of bulk RNA-seq data have provided plenty of support for the search of new targets or new therapeutics (Liu et al., 2018; Chen et al., 2022). However, the DEGs from bulk RNA-seq could only represent the average expression levels of the genes. Over the last decade, scRNA-seq technology has been increasingly accepted to be a cardinal method which could assist the gene annotation of the various biological roles at the single-cell level (Ren et al., 2021). Hence, in our study, we identified the DEGs of seven cell types between normal and tumor cells in breast cancer. With these genes, we further filtered prognosis-related genes and developed a prognostic signature to facilitate the prognosis prediction in clinical practice.

We envisaged that tumor and normal cells displayed distinct cell landscapes because of different gene expressions. Cell ratios were different between tumor and normal samples which may support this concept. Compared with other types of cancer, the immune cell ratios were lower in TNBC, which might be the reason for the poorer prognosis of TNBC patients (Mori et al., 2017). In most, if not all, tumor samples, the ratio of immune cells such as macrophages, T cells, and B cells increased dramatically compared to normal samples. It indicated that immune cells were recruited into the tumor environment during the multistep transformation of normal tissues into malignancies. The question now is why these immune cells fail to identify tumor cells or how these tumor cells evaded the destruction by immune cells. It might be rationalized by the concept of immune checkpoints. Immune checkpoint inhibitors (ICIs) which could change or reverse immune suppression (Wilky, 2019) have been commonly used for chemotherapeutic treatments for multiple types of cancers including breast cancer (Santa-Maria and Nanda, 2018). Hence, we also assessed the performance of the signature in therapeutic responses to ICIs in bulk RNA-seq data. Patients of the high-risk group were more sensitive to ICIs, which might be due to the lower immune infiltration and immunosuppression.

The DEGs between normal and tumor cells identified from the scRNA-seq data were further incorporated into the bulk data to filter prognosis-related genes. Only 163 genes met the criteria and were used to construct a prognostic signature through LASSO. The efficacy of the signature was significant in two independent cohorts. The AUCs of the united nomogram could also add new methods for clinical practice. The 18 genes in the signature merit further investigation due to the fact that they are differentially expressed at a single-cell level and hold important implications for prognosis. Notably, many genes were differentially expressed in virtually all cell types, such as ANXA5, which were downregulated in almost all tumor cell types. Moreover, virtually, all signature genes that were differentially expressed at a single-cell level were also differentially expressed at the bulk level, which could add evidence for the importance of these genes.

GSEA is a method that could help us detect subtle changes of the pathways between different groups (Subramanian et al., 2005). At the single-cell level, enrichment pathways between normal and cancer cells displayed a distinct landscape. For instance, pathways such as E2F targets and G2M checkpoint were more enriched in epithelial cells of cancer. Moreover, the DNA repair pathway was enriched more in virtually all cell types of cancer. At the bulk level, the pathways of the high-risk group were mainly enriched in metabolism-related pathways such as histidine metabolism and starch and sucrose metabolisms, which indicated the active metabolism of the mitochondria. This might suggest a higher degree of malignancy in the high-risk group and add evidence for mitochondria as the target in cancer treatment (Liu and Shi, 2020). In the low-risk group, immune-related pathways such as primary immunodeficiency were enriched, which might suggest that immune infiltration occurred more in low-risk samples. Moreover, although the evidence that the intestinal immune network for IgA production which was enriched in the low-risk group is still fragmentary, the functions of intestinal microorganisms in the resistance to tumors are still far beyond our reach and merit further investigation.

As to the biological roles and properties of the genes in the signature, several genes were annotated. For instance, it has been reported that a high ARID1B expression is associated with an increased histological grade and a decreased 5-year disease-free survival rate (Shao et al., 2015). In our results, ARID1B was upregulated in stem cells. Hence, it is reasonable to speculate that high ARID1B might contribute to the malignancy of tumors. Notably, the expression of ARID1B in the bulk RNA-seq data was inconsistent with that of the scRNA-seq data. We speculate that ARID1B might be differentially expressed in different molecular subtypes, which needs more evidence from experiments. Notably, ACSL1 was downregulated in the tumor macrophage in our study. Blocking ACSL1 in TNBC cells markedly suppressed the secretion of the granulocyte–macrophage colony-stimulating factor (GM-CSF) (Thomas et al., 2019). Also, it was reported that TNF-α prompts an M1-like phenotypic shift in the monocytes through ACSL1 (Al-Rashed et al., 2019). Moreover, macrophages account for larger ratios in cancer tissues. Therefore, ACSL1 might be a reflection of M1 macrophages, and M2 macrophages might be more prevalent in cancer tissues, especially in TNBC. In our signature, there are many genes whose functions in breast cancer have not been annotated and remain to be identified. For instance, OR51E1 was highly expressed in stem cells of cancer, and it contributed to an increased risk score in our signature. Hence, OR51E1 is worth a further study as an oncogene.

Taken together, these various lines of results indicate the importance of the signature and the genes in the signature. The genes might be important for understanding the conversion of normal cells into tumor cells. Such knowledge that exploits these differences may provide, in turn, opportunities to develop novel therapies. We also added evidence for the heterogeneity of the patients and the importance of immune infiltration. However, our research was conducted based on transcriptomics data. Proteomics data are also needed because proteins are the main executors of functions, and their expression levels are not always consistent with RNA expression. Hereby, selective co-targeting of many oncogenes based on the multi-omics detection of every patient and various types of immunotherapies would be the most efficient method for precision medicine in the future.

## Conclusion

DEGs between normal and tumor cells were identified through single-cell transcriptome data. The signature constructed with these DEGs could stratify patients efficiently. The signature was also closely correlated with immune infiltration and TMB. Nearly all the genes in the signature were also differentially expressed at the bulk RNA-seq level.

## Data Availability

Publicly available datasets were analyzed in this study. The names of the repository/repositories and accession number(s) can be found in the article/Supplementary Material.
