# Supplementary figures and images for: Identification of differentially expressed genes at the single-cell level and prognosis prediction through bulk RNA sequencing data in breast cancer
Source: Front Genet. 2022 Sep 16;13:979829. doi: 10.3389/fgene.2022.979829 (PMC9523223; doi:10.3389/fgene.2022.979829)

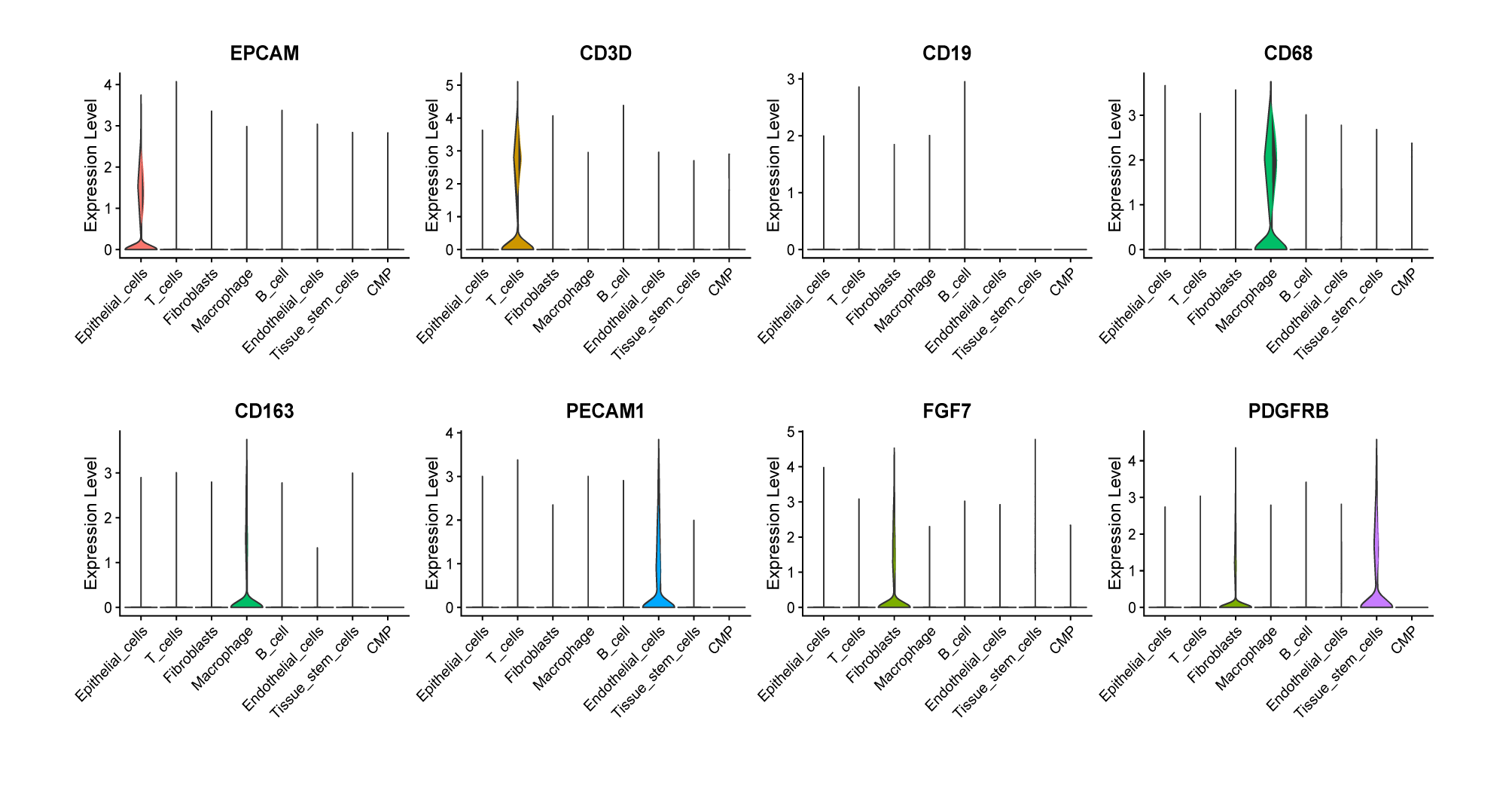

Supplement: Supplementary file 3 [file Image2.TIF]

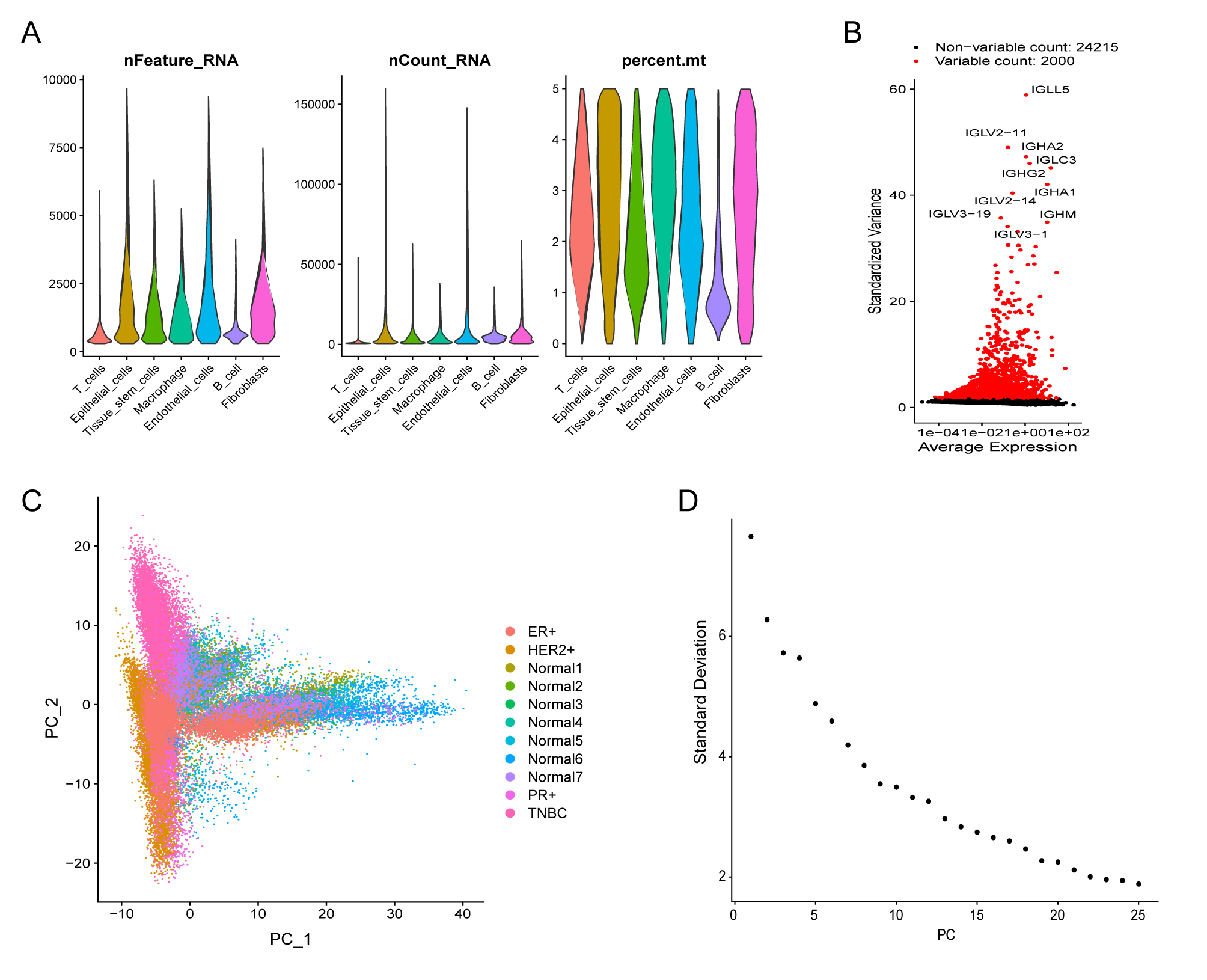

Supplement: Supplementary file 4 [file Image1.TIF]
